# Supplementary figures and images for: Live imaging of echinoderm embryos to illuminate evo-devo
Source: Front Cell Dev Biol. 2022 Sep 15;10:1007775. doi: 10.3389/fcell.2022.1007775 (PMC9521734; doi:10.3389/fcell.2022.1007775)

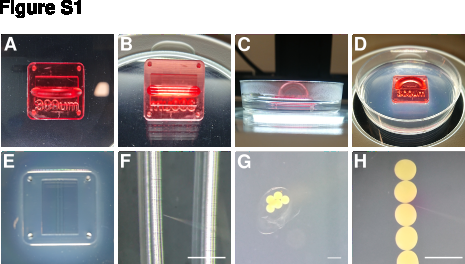

Supplement: Supplementary file 2 [file Image1.TIFF]
