# Supplementary material for: Live imaging of echinoderm embryos to illuminate evo-devo
Source: Front Cell Dev Biol. 2022 Sep 15;10:1007775. doi: 10.3389/fcell.2022.1007775 (PMC9521734; doi:10.3389/fcell.2022.1007775)
Supplement: Supplementary file 4 [file Presentation1.PDF]

Supplementary Methods

Live imaging of *Crepidula atrasolea* embryos

Crepidula atrasolea colonies were maintained in the lab, and zygotes were collected as previously described (Henry et al., 2017). Briefly, adult females harboring broods of embryos were gently detached from their substrate, allowing the collection of embryos from underneath them (Henry et al., 2017): several bags containing clutches of embryos can be found underneath a brooding female (Henry et al., 2017); (video tutorials available at https://www.life.illinois.edu/henry/tools.html). Each bag contains a clutch of embryos that were laid approximately at the same time. All embryos were raised in 0.22 µm filtered sea water (FSW) with the addition of 60 µg/ml Penicillin G sodium salt (Millipore Sigma, P3032) and 200 µg/ml Streptomycin sulfate salt (Millipore Sigma, S1277).
mRNAs were synthesized with the mMessage mMachine SP6 Transcription Kit (Invitrogen, AM1340). Injection dishes were prepared by placing a 3D printed stamp (Truchado-Garcia et al., 2018) (design downloadable at https://www.life.illinois.edu/henry/3Dembryos_index.html) onto molten agarose (2% agarose in FSW), letting the agarose solidify, removing the stamp and then filling the dish with PS-FSW. Bags containing uncleaved embryos (zygotes) were transferred to an agarose injection dish and opened with forceps to gently free the embryos from the bags and align them for injections (Supplementary Figure S1). Zygotes were injected with mRNAs to label membranes (lck-mCitrine, 100 ng/µl) and nuclei (H2B-RFP, 400 ng/µl). Injected embryos were incubated in the agarose dish at 27°C until the 4-cell stage and then mounted on a BSA coated glass bottom dish (MatTek, P35G-1.5-14-C). No medium was used to immobilize the embryos: the glass bottom part of the dish was covered with a coverslip and sealed with vaseline. This creates a 600 µm deep chamber in which capillarity prevents the embryos from moving, until they develop cilia. Additional FSW was added in the dish, to help with temperature control. Embryos were imaged on an inverted Leica Sp8 confocal microscope (20X objective, NA 0.7, 27°C controlled temperature) for 15 hours with z-stacks (0.91 µm z-step) acquired every 15 min. Datasets were 3D rendered using Imaris 6.4 (Bitplane).
A step by step protocol is provided below:

Materials		Tips
**Injection set up:**	Microinjector (e.g. Narishige IM-400).<br><br>High magnification stereoscope (e.g. Leica MDG41).<br><br>Micromanipulator (e.g. Narishige MN-4 and MMO-4).	
**Gelatin**	Prepare a 5X stock solution of gelatin: 0.5% Knox unflavored gelatin and 0.19 % formaldehyde in dH20	Make sure to dissolve the gelatin before adding formaldehyde to the solution. Both the stock and 1X gelatin solution can be stored at RT for months.
**Gelatin coated glass transfer pipettes:**	Pull, break, flame polish and gelatine coat glass transfer pipettes (e.g. VWR 14673-043) to handle the embryos.	*C. atrasolea* embryos are very delicate, so it is important that transfer pipettes have an opening diameter slightly larger than the embryos, flame polished to remove any sharp edges and gelatin coated. If gentle pipetting of embryos is a challenge, more delicate handling of the embryos can be achieved via mouth pipetting.
**Needles:**	Pull needles, using a borosilicate capillary with filament and 1 mm external diameter (MPI TW-100). Using a Sutter Instrument P-1000 needle puller we use the following settings: Heat	If possible, pull needles shortly before injection, to avoid dust or other particles from depositing inside the needle and

= Ramp +10; Pull = 90; Velocity = 80; Delay = 90; Pressure 200; Delay mode: active.

possibly causing clogging during injection.

Injection dish:

Pour molten 2% agarose in FSW into a 60 mm petri dish.

Place a 3D printed stamp on the agarose and let it solidify.

Remove the stamp carefully and check that the injection grooves are intact.

Cover the agarose with PS-FSW.

Prepare injection dishes on the same day you plan to inject. Make sure to add PS-FSW, otherwise the agarose will dry and shrink. Rinse once with fresh PS-FSW before starting transferring embryos to the dish.

Scratch dish:

Gelatin coat a 35 mm petri dish.

Scratch a few lines on the plastic with a forcep: this will be useful to break open the injection needle.

mRNAs:

To synthesize the mRNA for injection, in this case mRNA coding for membrane bound mCitirine and H2B-RFP, follow these steps:

Linearize pCS2-lck-mCitrine and pCS2-H2BRFP plasmids by digesting with NotI and clean up the digest using the Qiagen PCR clean up kit.

Run the linearized plasmid on a gel to make sure the plasmids are fully linearized.

Use mMessage mMachine mRNA synthesis kit with the linearized plasmid as template.

Precipitate mRNA with Phenol Chloroform.

Re-suspend mRNA in 15 µl of nuclease free water for a 20 µl mMessage machine reaction. Quantify mRNA concentration, e.g. with a Nanodrop, and run a gel to check for high

For best results, digest 10 µg plasmid overnight and then elute the linearized plasmid in 20 µl of nuclease free water.

For mRNA synthesis with mMessage mMachine SP6 kit, we have best results incubating the reaction overnight at 30°C, instead of the recommended 2h at 37°C.

molecular weight bands and no degradation.

Preserve mRNA stock at a concentration of 1-2 µg/µl at -20C.

Injection mix:

Prepare 2 µl mix working solution of 50-100 ng/µl mCitrine mRNA and 200-400 ng/µl H2B-RFP mRNA in 0.5% Phenol Red solution (P0290, Sigma).

Spin the injection mix for 2min at 16K g.

Prepare an injection mix immediately prior to injecting. Phenol red is necessary to monitor injections, because the embryos are yolky and dark. Dilute the stock into 0.5% Phenol Red to a maximum of 1:1 dilution of the Phenol Red.

Zygotes (uncleaved):

Zygotes are obtained by detaching adult females from their substrate and collecting broods: each clutch of embryos is enclosed in a bag.

Select bags containing uncleaved embryos.

At 25-27 °C the zygote stage lasts for approximately 6 hours. All embryos within a bag were laid at the same time. If a few embryos within the bag are at the 2-cell stage, the other ones will divide soon. Injecting such embryos may result in uneven labeling.

Matek dish:

P35G-1.5-14-C

Vaseline:

Just regular vaseline from your favorite shop.

Procedure: injection

Tips

Load needles:

Place 0.5 µl of injection mix on the back of a needle that is held vertically.

Wait 2-5 min for the injection mix to descend the needle via capillary action.

Position embryos :

Place one or two bags of embryos inside the injection dish.

Open the bag using forceps and gently free the embryos.

Line up the embryos in the groove of the injection dish using a gelatin coated glass pipette or eyelash tool.

Position scratch dish:

Gently move away the injection dish.

Fill the scratched dish with FSW and position it on the stereoscope.

Break needle:

Insert the loaded needle in the microinjector holder.

Tip: To inject inside the agarose dish, it is best to position the needle at a 45° angle.

Adjust the injector so that a small positive pressure is applied to the needle. Using a Narishige IM-400 we set balance pressure to 60-100 KPa.

Position the needle in the middle of the microscope field of view.

Break the needle by bringing into contact with the ridge of a scratch in your gelatin coated dish.

Tip: Break the needle as gently as possible: a narrow tip needle will penetrate the yolky snail embryo without inflicting damage. However, narrow needles

Make sure the injection mix is flowing and that you can see the phenol red.

also clog up more easily, making injections rather frustrating. The optimal needle opening needs to be determined empirically.

Inject!

Move away the scratch dish and position the injection dish containing the embryos onto the stereoscope.

Position the needle so that it is at a 45° angle and the tip is in the center of the field of view.

Make sure the injection mix is flowing.

Inject each embryo by gently pressing on its membrane.

Remove the needle once you see a small puff of phenol red that disperses quickly. If you see a lingering red region, you have injected too much.

For best results, stay very close to the surface of the embryo, as going deeper will result in irreparable damage, with yolk flowing out of the embryo.

For these embryos, less is more: inject as little as possible. You are aiming for a small puff of phenol red inside the embryo that disperses quickly. Injecting larger volumes often results in the embryo bursting open later on.

Clean up:

Remove all damaged embryos.

Leave the injected embryos untouched until they reach the desired stage (e.g. 4-cell).

These embryos will not survive in agarose for a longer time period: if they are to be raised until after cleavage stages they need to be transferred to BSA coated dishes.

Procedure: mounting		Tips
**Prepare MatTek dish:**	Prepare a MatTek dish chamber by brushing a thin layer of vaseline on the plastic portion of the MatTek dish, just around the base coverslip.<br><br>Coat the glass bottom part of the MatTek dish with 5% BSA in H_2O for 2-5 min.<br><br>Wash out the BSA with 500 µl of FSW three times.<br><br>Fill the inner chamber of the MatTek dish with 250 µl of PS-FSW.	BSA coating of the MatTek dish is necessary because the embryos are denuded of their bag and will stick to uncoated glass
**Transfer embryos:**	Transfer a maximum of 6 embryos to the MatTek dish, placing them in the center of the base coverslip.	This method does not allow orienting of the mounted embryos. At 4-cell stage, the embryos will fall with either the vegetal or the animal side facing the coverslip, with 50% chance of getting the desired orientation.
**Seal embryos:**	Gently place a 22X22 coverslip on top of the PS-FSW containing the embryos and push it down slowly so that excess water is pushed out of the chamber while the embryos remain somewhat centered in the dish.<br><br>Fill the MatTek dish with 2 ml of PS-FSW.	When pushing the top coverslip down, make sure that a uniform layer of vaseline has sealed the coverslip in place. If in doubt, add a thick layer of vaseline all around the top coverslip. Adding water to the MatTek dish after mounting helps maintain the desired temperature in the dish.

Procedure: imaging

Tips

The mounted embryos are now ready to be imaged. Settings will vary depending on the scope of imaging. Here we will refer to the settings we used to image multiple embryos for up to 16 hours.

<u>Set temperature:</u>	One hour before imaging, start the cooling of the temperature controlled stage.	This allows the stage to be at temperature when starting imaging, thereby reducing drift during the acquisition
<u>Adjust imaging settings:</u>	Set-up the imaging software as needed. In this case we used a Leica Sp8 inverted confocal with settings:<br>- time-lapse<br>- multi-positioning<br>- z-stacks<br>- two channels: YFP (excitation laser: 514 nm; acquisition range: 520 nm - 547 nm) and RFP (excitation laser: 552; acquisition range: 568 nm - 700 nm)<br>- Resonant scanner 8000Hz<br>- Line average 3<br>- Frame average 1<br>- 20X objective, 0.70 NA<br>- Zoom 1.25<br>- Pinhole at 1.20 AU<br>- Z-step: 0.91 µm	
<u>Set up imaging:</u>	Once the MatTek dish is placed on the microscope stage, find the embryos to image and mark their position in the software.<br><br>Set an appropriate z-stack for each position<br><br>Set duration of acquisition (e.g. 16 hours) and timeframe (e.g. 15 min)<br><br>Hit the start button!	Make sure the z-stack starts from below the coverslip and to set the z-stack a bit bigger than what seems necessary. This helps avoid the sample moving out of the set z-stack because of morphogenesis or stage drift.

References

Henry, J. Q., Lesoway, M. P., Perry, K. J., Osborne, C. C., Shankland, M., and Lyons, D. C. (2017). Beyond the sea: Crepidula atrasolea as a spiralian model system. *Int. J. Dev. Biol.* 61, 479–493.

Truchado-Garcia, M., Harland, R. M., and Abrams, M. J. (2018). 3D-printable tools for developmental biology: Improving embryo injection and screening techniques through 3D-printing technology. *bioRxiv*. doi: 10.1101/376657.

https://www.life.illinois.edu/henry/tools.html : *C. atrasolea* handling video tutorials.

https://www.life.illinois.edu/henry/3Dembryos_index.html: injection stamp stl file.
